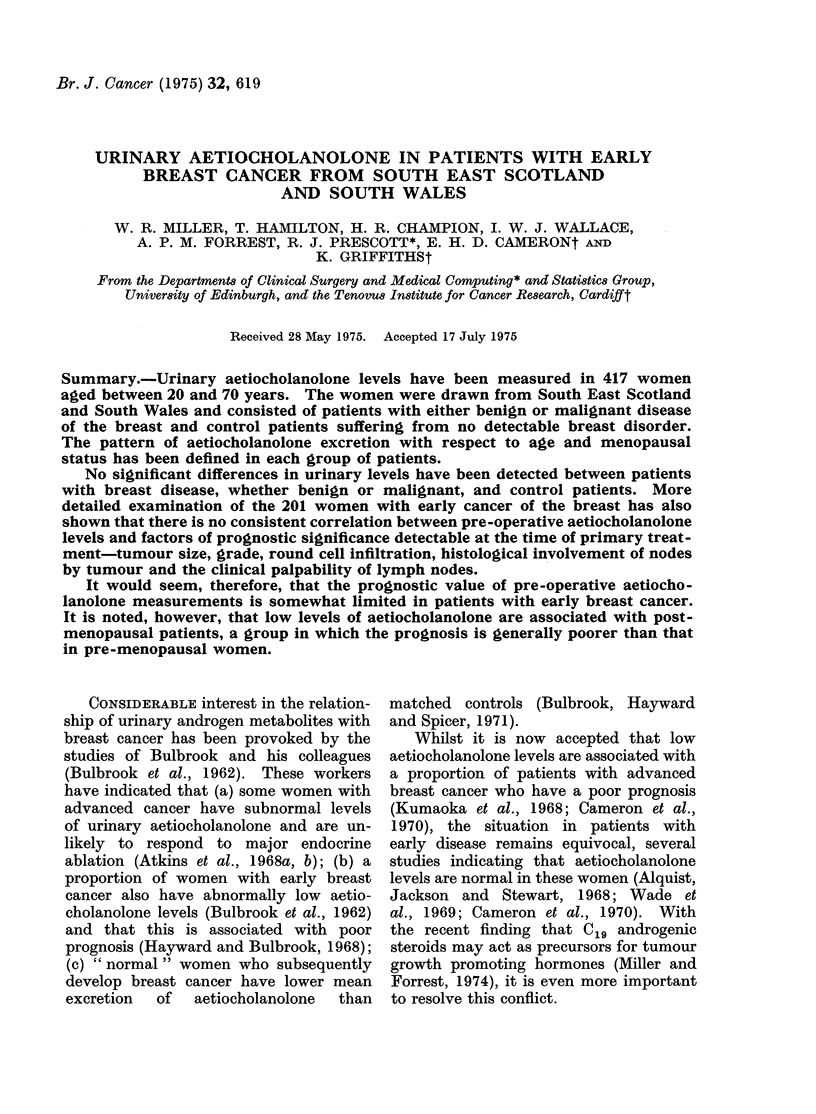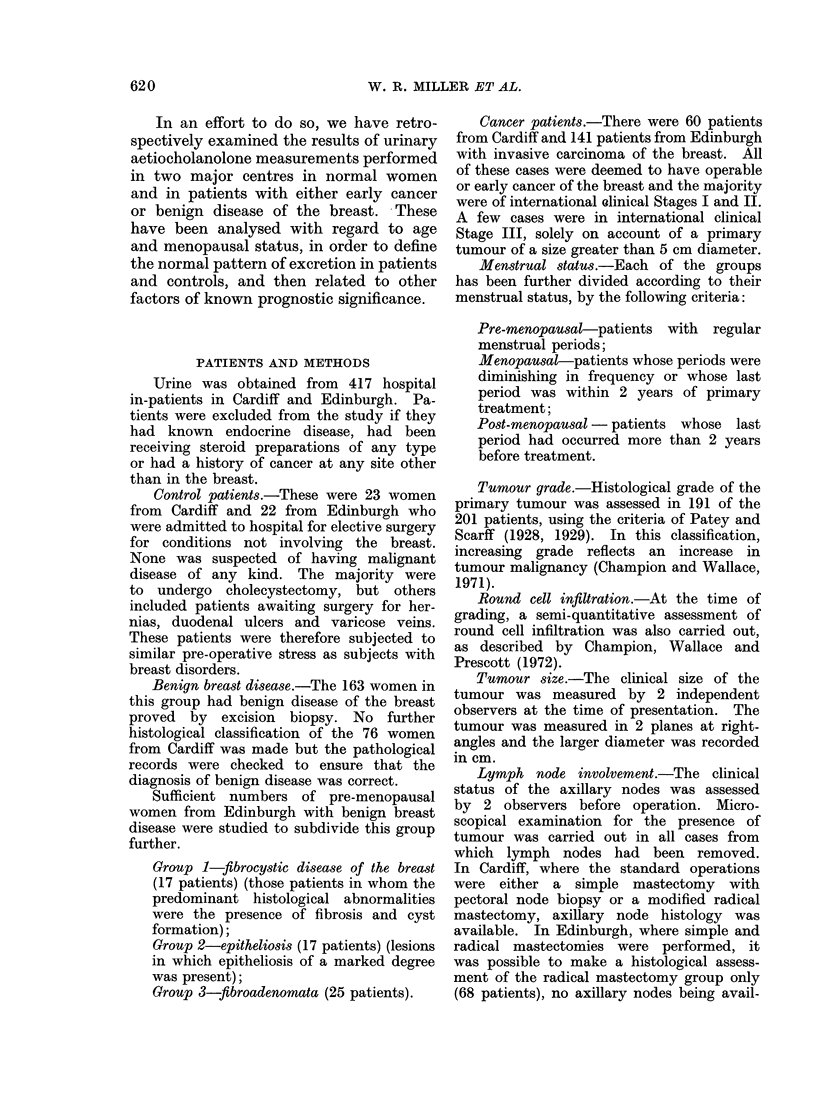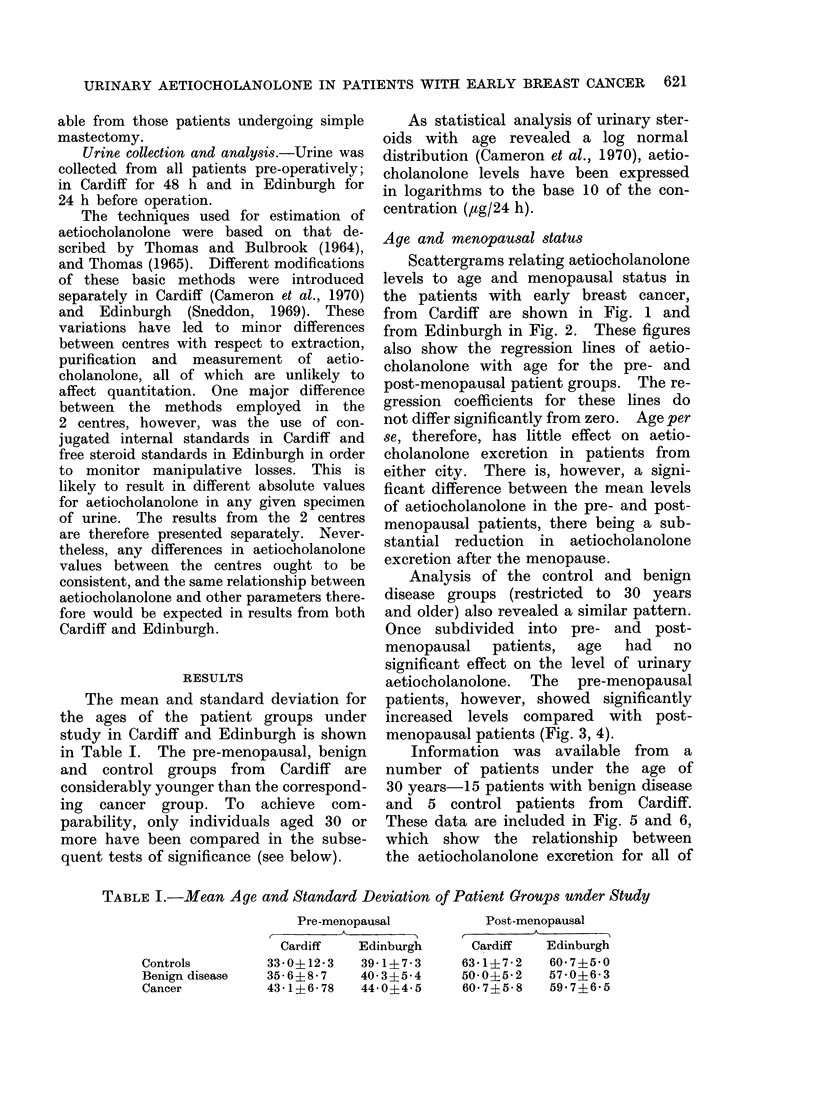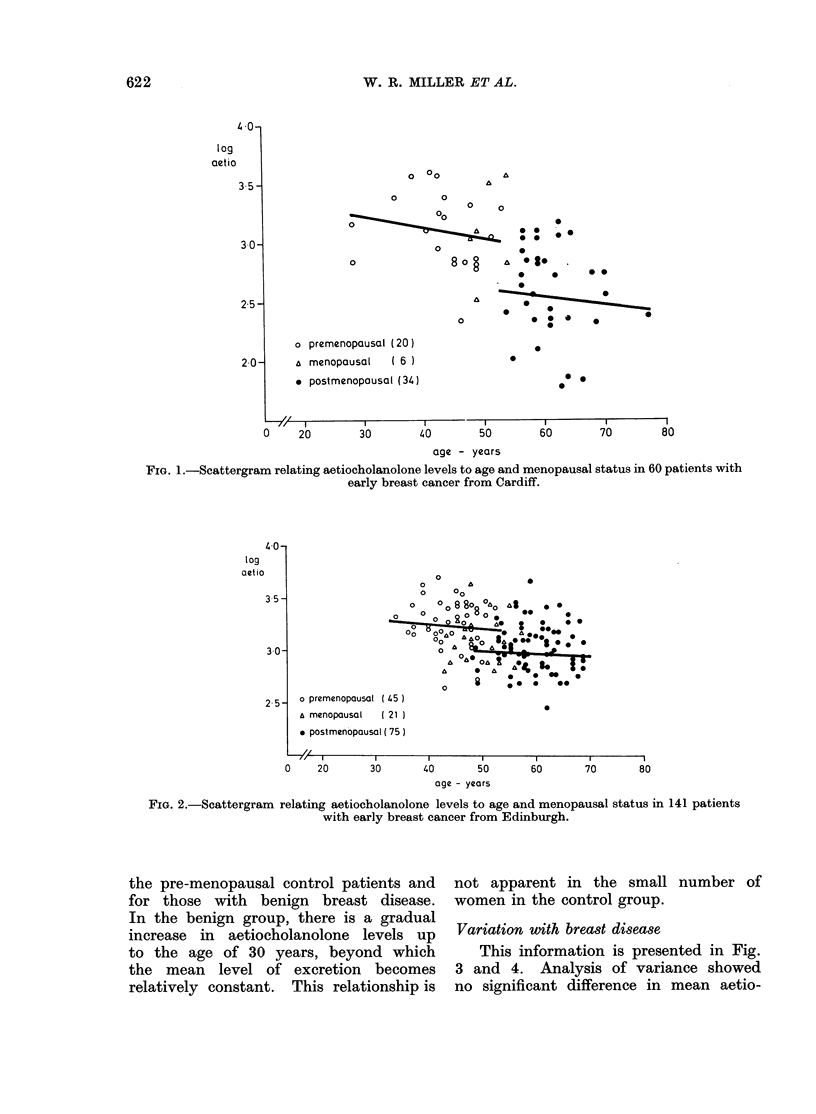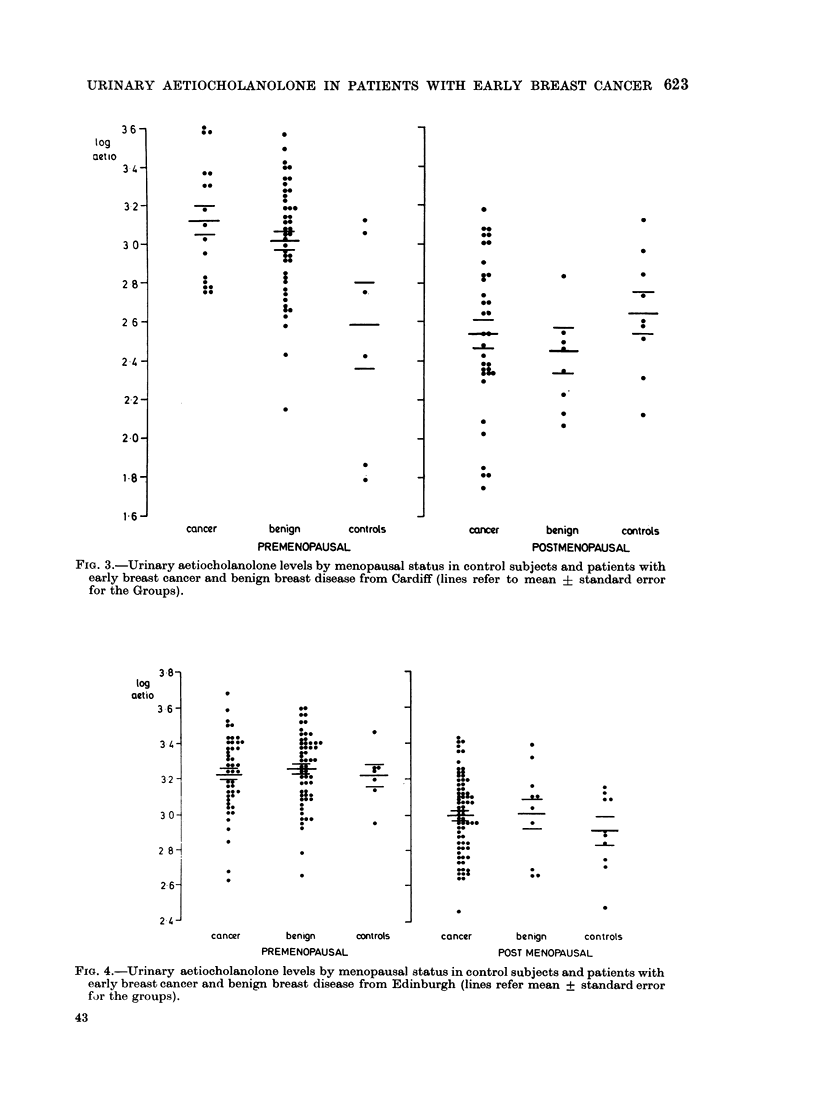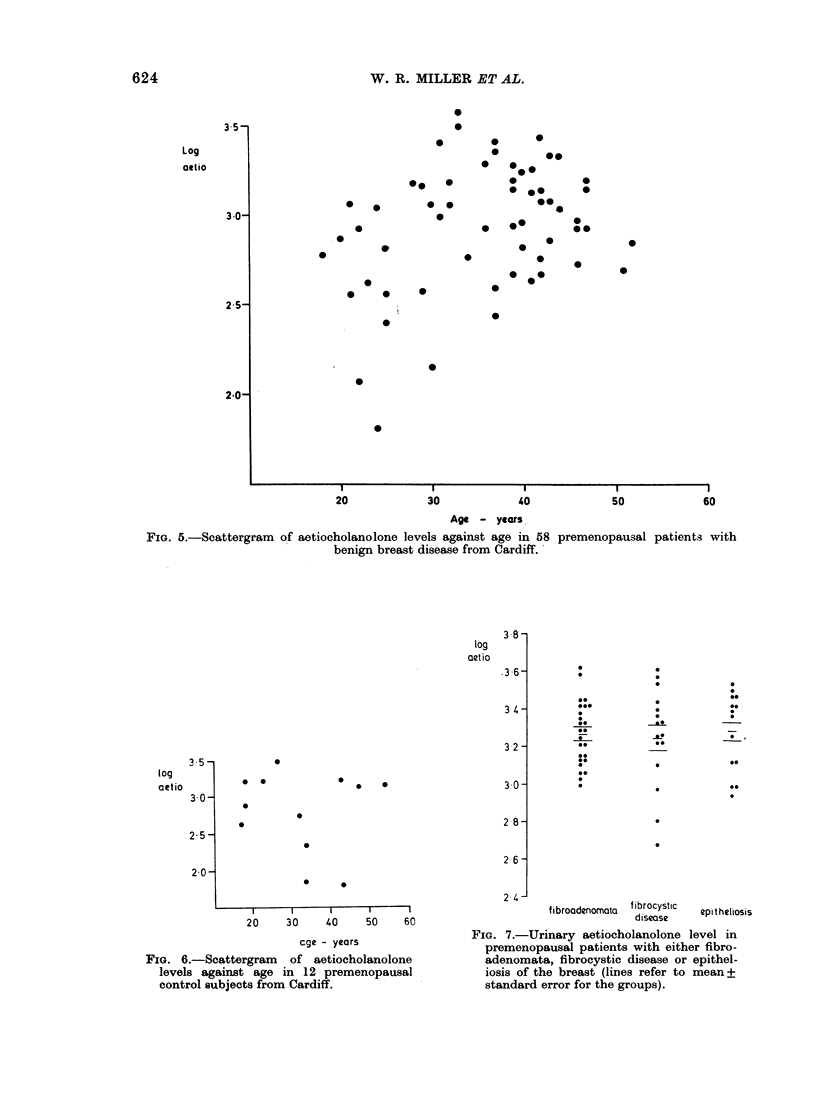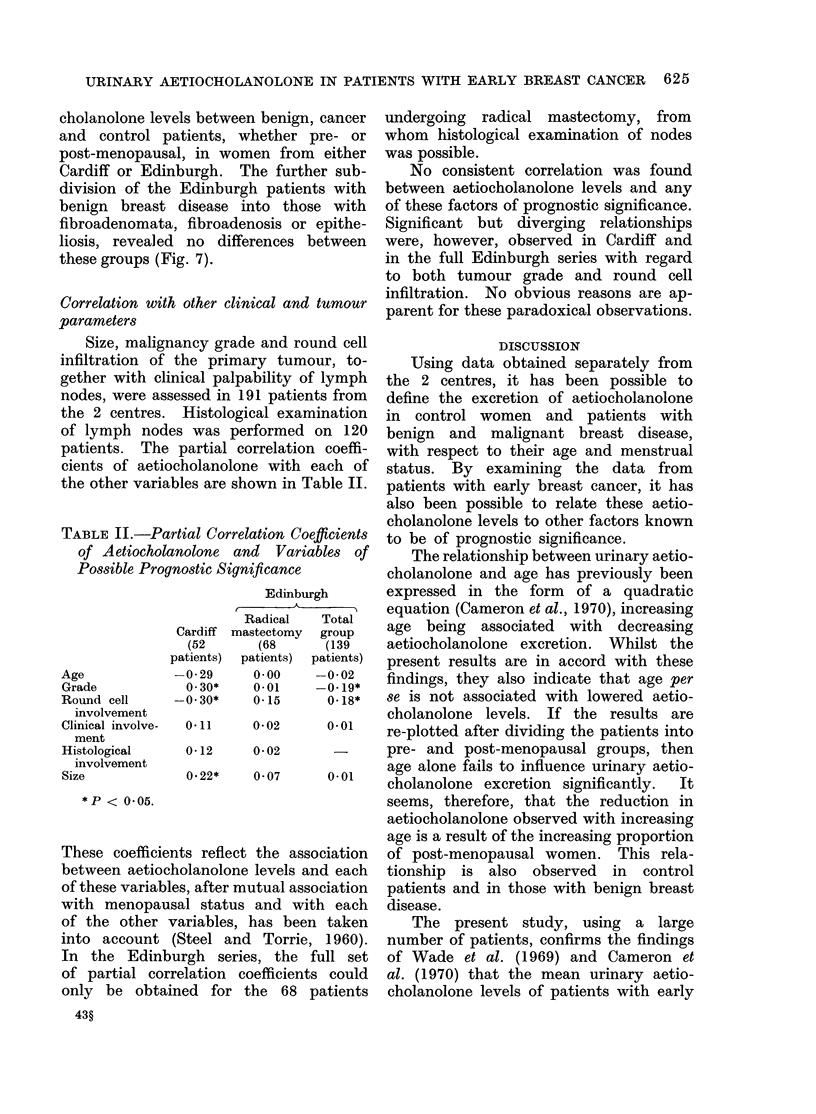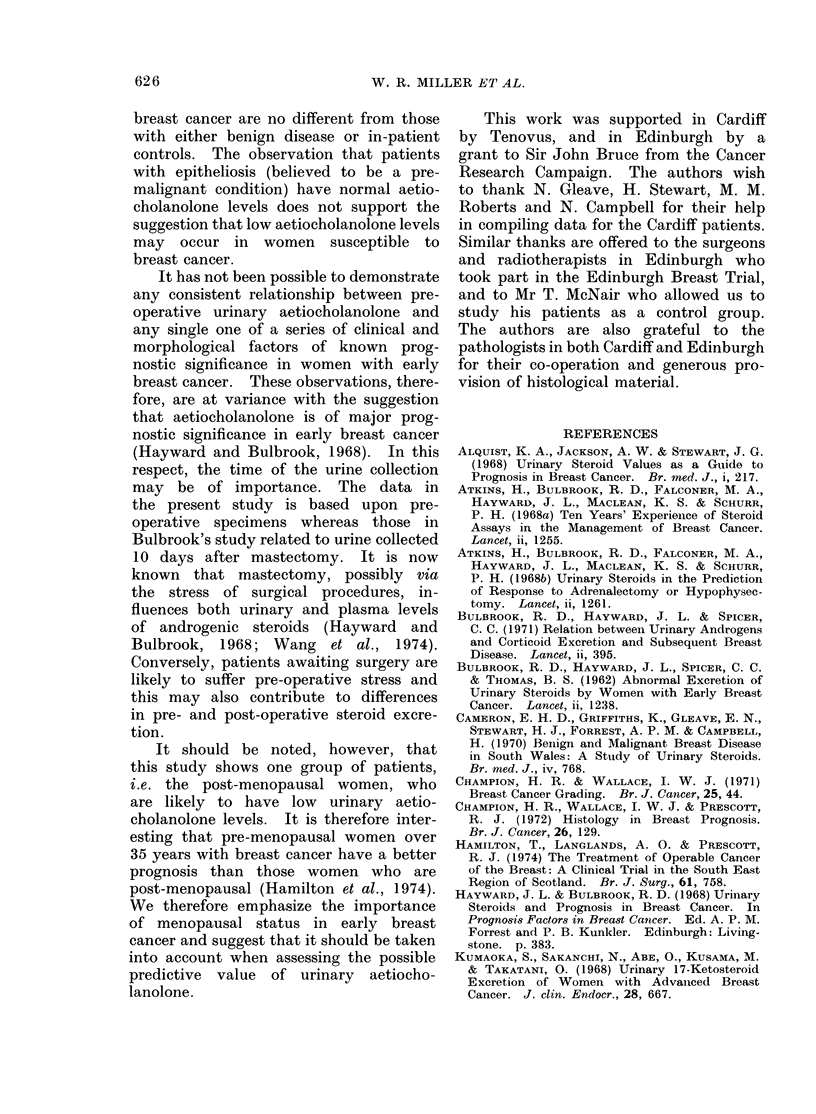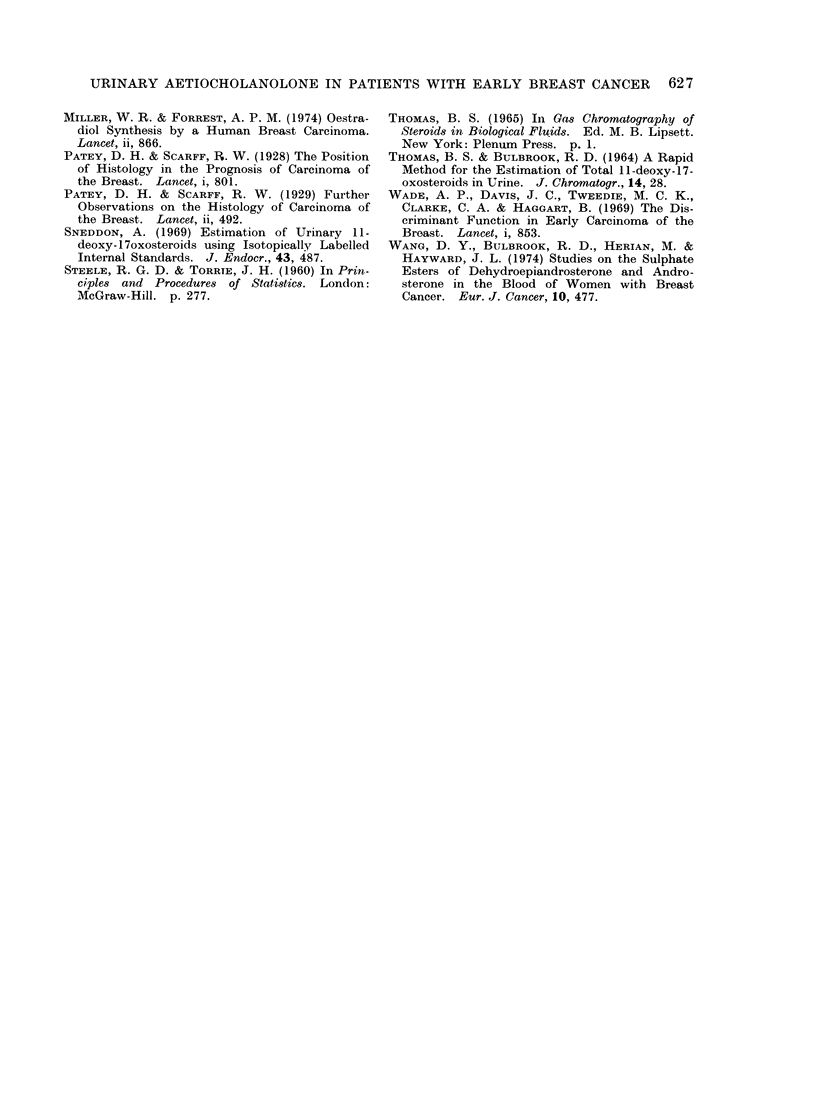# Urinary aetiocholanolone in patients with early breast cancer from South East Scotland and South Wales.

**DOI:** 10.1038/bjc.1975.269

**Published:** 1975-11

**Authors:** W. R. Miller, T. Hamilton, H. R. Champion, I. W. Wallace, A. P. Forrest, R. J. Prescott, E. H. Cameron, K. Griffiths

## Abstract

Urinary aetiocholanolone levels have been measured in 417 women aged between 20 and 70 years. The women were drawn from South East Scotland and South Wales and consisted of patients with either benign or malignant disease of the breast and control patients suffering from no detectable breast disorder. The pattern of aetiocholanolone excretion with respect to age and menopausal status has been defined in each group of patients. No significant differences in urinary levels have been detected between patients with breast disease, whether benign or malignant, and control patients. More detailed examination of the 201 women with early cancer of the breast has also shown that there is no consistent correlation between pre-operative aetiocholanolone levels and factors of prognostic significance detectable at the time of primary treatment-tumour size, grade, round cell infiltration, histological involvement of nodes by tumour and the clinical palpability of lymph nodes. It would seem, therefore, that the prognostic value of pre-operative aetiocholanolone measurements is somewhat limited in patients with early breast cancer. It is noted, however, that low levels of aetiocholanolone are associated with post-menopausal patients, a group in which the prognosis is generally poorer than that in pre-menopausal women.


*Br. J. Cancer* (1975) **32,** 619

### URINARY AETIOCHOLANOLONE IN PATIENTS WITH EARLY BREAST CANCER FROM SOUTH EAST SCOTLAND AND SOUTH WALES

W. R. MILLER, T. HAMILTON, H. R. CHAMPION, I. W. J. WALLACE, A. P. M. FORREST, R. J. PRESCOTT*, E. H. D. CAMERON† AND K. GRIFFITHS†

*From the Departments of Clinical Surgery and Medical Computing* and Statistics Group, University of Edinburgh, and the Tenovus Institute for Cancer Research, Cardiff†*

Received 28 May 1975. Accepted 17 July 1975

**Summary.—Urinary aetiocholanolone levels have been measured in 417 women aged between 20 and 70 years. The women were drawn from South East Scotland and South Wales and consisted of patients with either benign or malignant disease of the breast and control patients suffering from no detectable breast disorder. The pattern of aetiocholanolone excretion with respect to age and menopausal status has been defined in each group of patients.**

**No significant differences in urinary levels have been detected between patients with breast disease, whether benign or malignant, and control patients. More detailed examination of the 201 women with early cancer of the breast has also shown that there is no consistent correlation between pre-operative aetiocholanolone levels and factors of prognostic significance detectable at the time of primary treatment—tumour size, grade, round cell infiltration, histological involvement of nodes by tumour and the clinical palpability of lymph nodes.**

**It would seem, therefore, that the prognostic value of pre-operative aetiocholanolone measurements is somewhat limited in patients with early breast cancer. It is noted, however, that low levels of aetiocholanolone are associated with post-menopausal patients, a group in which the prognosis is generally poorer than that in pre-menopausal women.**

CONSIDERABLE interest in the relationship of urinary androgen metabolites with breast cancer has been provoked by the studies of Bulbrook and his colleagues (Bulbrook *et al.*, 1962). These workers have indicated that (a) some women with advanced cancer have subnormal levels of urinary aetiocholanolone and are unlikely to respond to major endocrine ablation (Atkins *et al.*, 1968*a*, *b*); (b) a proportion of women with early breast cancer also have abnormally low aetiocholanolone levels (Bulbrook *et al.*, 1962) and that this is associated with poor prognosis (Hayward and Bulbrook, 1968); (c) " normal " women who subsequently develop breast cancer have lower mean excretion of aetiocholanolone than matched controls (Bulbrook, Hayward and Spicer, 1971).

Whilst it is now accepted that low aetiocholanolone levels are associated with a proportion of patients with advanced breast cancer who have a poor prognosis (Kumaoka *et al.*, 1968; Cameron *et al.*, 1970), the situation in patients with early disease remains equivocal, several studies indicating that aetiocholanolone levels are normal in these women (Alquist, Jackson and Stewart, 1968; Wade *et al.*, 1969; Cameron *et al.*, 1970). With the recent finding that $C_{19}$ androgenic steroids may act as precursors for tumour growth promoting hormones (Miller and Forrest, 1974), it is even more important to resolve this conflict.

In an effort to do so, we have retrospectively examined the results of urinary aetiocholanolone measurements performed in two major centres in normal women and in patients with either early cancer or benign disease of the breast. These have been analysed with regard to age and menopausal status, in order to define the normal pattern of excretion in patients and controls, and then related to other factors of known prognostic significance.

### PATIENTS AND METHODS

Urine was obtained from 417 hospital in-patients in Cardiff and Edinburgh. Patients were excluded from the study if they had known endocrine disease, had been receiving steroid preparations of any type or had a history of cancer at any site other than in the breast.

*Control patients.*—These were 23 women from Cardiff and 22 from Edinburgh who were admitted to hospital for elective surgery for conditions not involving the breast. None was suspected of having malignant disease of any kind. The majority were to undergo cholecystectomy, but others included patients awaiting surgery for hernias, duodenal ulcers and varicose veins. These patients were therefore subjected to similar pre-operative stress as subjects with breast disorders.

*Benign breast disease.*—The 163 women in this group had benign disease of the breast proved by excision biopsy. No further histological classification of the 76 women from Cardiff was made but the pathological records were checked to ensure that the diagnosis of benign disease was correct.

Sufficient numbers of pre-menopausal women from Edinburgh with benign breast disease were studied to subdivide this group further.

*Group 1—fibrocystic disease of the breast* (17 patients) (those patients in whom the predominant histological abnormalities were the presence of fibrosis and cyst formation);

*Group 2—epitheliosis* (17 patients) (lesions in which epitheliosis of a marked degree was present);

*Group 3—fibroadenomata* (25 patients).

*Cancer patients.*—There were 60 patients from Cardiff and 141 patients from Edinburgh with invasive carcinoma of the breast. All of these cases were deemed to have operable or early cancer of the breast and the majority were of international clinical Stages I and II. A few cases were in international clinical Stage III, solely on account of a primary tumour of a size greater than 5 cm diameter.

*Menstrual status.*—Each of the groups has been further divided according to their menstrual status, by the following criteria:

*Pre-menopausal*—patients with regular menstrual periods;
*Menopausal*—patients whose periods were diminishing in frequency or whose last period was within 2 years of primary treatment;
*Post-menopausal* — patients whose last period had occurred more than 2 years before treatment.

*Tumour grade.*—Histological grade of the primary tumour was assessed in 191 of the 201 patients, using the criteria of Patey and Scarff (1928, 1929). In this classification, increasing grade reflects an increase in tumour malignancy (Champion and Wallace, 1971).

*Round cell infiltration.*—At the time of grading, a semi-quantitative assessment of round cell infiltration was also carried out, as described by Champion, Wallace and Prescott (1972).

*Tumour size.*—The clinical size of the tumour was measured by 2 independent observers at the time of presentation. The tumour was measured in 2 planes at right-angles and the larger diameter was recorded in cm.

*Lymph node involvement.*—The clinical status of the axillary nodes was assessed by 2 observers before operation. Microscopical examination for the presence of tumour was carried out in all cases from which lymph nodes had been removed. In Cardiff, where the standard operations were either a simple mastectomy with pectoral node biopsy or a modified radical mastectomy, axillary node histology was available. In Edinburgh, where simple and radical mastectomies were performed, it was possible to make a histological assessment of the radical mastectomy group only (68 patients), no axillary nodes being available from those patients undergoing simple mastectomy.

*Urine collection and analysis.*—Urine was collected from all patients pre-operatively; in Cardiff for 48 h and in Edinburgh for 24 h before operation.

The techniques used for estimation of aetiocholanolone were based on that described by Thomas and Bulbrook (1964), and Thomas (1965). Different modifications of these basic methods were introduced separately in Cardiff (Cameron *et al.*, 1970) and Edinburgh (Sneddon, 1969). These variations have led to minor differences between centres with respect to extraction, purification and measurement of aetiocholanolone, all of which are unlikely to affect quantitation. One major difference between the methods employed in the 2 centres, however, was the use of conjugated internal standards in Cardiff and free steroid standards in Edinburgh in order to monitor manipulative losses. This is likely to result in different absolute values for aetiocholanolone in any given specimen of urine. The results from the 2 centres are therefore presented separately. Nevertheless, any differences in aetiocholanolone values between the centres ought to be consistent, and the same relationship between aetiocholanolone and other parameters therefore would be expected in results from both Cardiff and Edinburgh.

### RESULTS

The mean and standard deviation for the ages of the patient groups under study in Cardiff and Edinburgh is shown in Table I. The pre-menopausal, benign and control groups from Cardiff are considerably younger than the corresponding cancer group. To achieve comparability, only individuals aged 30 or more have been compared in the subsequent tests of significance (see below).

As statistical analysis of urinary steroids with age revealed a log normal distribution (Cameron *et al.*, 1970), aetiocholanolone levels have been expressed in logarithms to the base 10 of the concentration ($\mu$g/24 h).

*Age and menopausal status*

Scattergrams relating aetiocholanolone levels to age and menopausal status in the patients with early breast cancer, from Cardiff are shown in Fig. 1 and from Edinburgh in Fig. 2. These figures also show the regression lines of aetiocholanolone with age for the pre- and post-menopausal patient groups. The regression coefficients for these lines do not differ significantly from zero. Age *per se*, therefore, has little effect on aetiocholanolone excretion in patients from either city. There is, however, a significant difference between the mean levels of aetiocholanolone in the pre- and post-menopausal patients, there being a substantial reduction in aetiocholanolone excretion after the menopause.

Analysis of the control and benign disease groups (restricted to 30 years and older) also revealed a similar pattern. Once subdivided into pre- and post-menopausal patients, age had no significant effect on the level of urinary aetiocholanolone. The pre-menopausal patients, however, showed significantly increased levels compared with post-menopausal patients (Fig. 3, 4).

Information was available from a number of patients under the age of 30 years—15 patients with benign disease and 5 control patients from Cardiff. These data are included in Fig. 5 and 6, which show the relationship between the aetiocholanolone excretion for all of

TABLE I.—*Mean Age and Standard Deviation of Patient Groups under Study*

| | Pre-menopausal | | Post-menopausal | |
|---|---|---|---|---|
| | Cardiff | Edinburgh | Cardiff | Edinburgh |
| Controls | 33·0±12·3 | 39·1±7·3 | 63·1±7·2 | 60·7±5·0 |
| Benign disease | 35·6±8·7 | 40·3±5·4 | 50·0±5·2 | 57·0±6·3 |
| Cancer | 43·1±6·78 | 44·0±4·5 | 60·7±5·8 | 59·7±6·5 |

FIG. 1.—Scattergram relating aetiocholanolone levels to age and menopausal status in 60 patients with early breast cancer from Cardiff.

FIG. 2.—Scattergram relating aetiocholanolone levels to age and menopausal status in 141 patients with early breast cancer from Edinburgh.

the pre-menopausal control patients and for those with benign breast disease. In the benign group, there is a gradual increase in aetiocholanolone levels up to the age of 30 years, beyond which the mean level of excretion becomes relatively constant. This relationship is not apparent in the small number of women in the control group.

*Variation with breast disease*

This information is presented in Fig. 3 and 4. Analysis of variance showed no significant difference in mean aetio-

FIG. 3.—Urinary aetiocholanolone levels by menopausal status in control subjects and patients with early breast cancer and benign breast disease from Cardiff (lines refer to mean ± standard error for the Groups).

FIG. 4.—Urinary aetiocholanolone levels by menopausal status in control subjects and patients with early breast cancer and benign breast disease from Edinburgh (lines refer mean ± standard error for the groups).

FIG. 5.—Scattergram of aetiocholanolone levels against age in 58 premenopausal patients with benign breast disease from Cardiff.

FIG. 6.—Scattergram of aetiocholanolone levels against age in 12 premenopausal control subjects from Cardiff.

FIG. 7.—Urinary aetiocholanolone level in premenopausal patients with either fibroadenomata, fibrocystic disease or epitheliosis of the breast (lines refer to mean ± standard error for the groups).

cholanolone levels between benign, cancer and control patients, whether pre- or post-menopausal, in women from either Cardiff or Edinburgh. The further sub-division of the Edinburgh patients with benign breast disease into those with fibroadenomata, fibroadenosis or epitheliosis, revealed no differences between these groups (Fig. 7).

*Correlation with other clinical and tumour parameters*

Size, malignancy grade and round cell infiltration of the primary tumour, together with clinical palpability of lymph nodes, were assessed in 191 patients from the 2 centres. Histological examination of lymph nodes was performed on 120 patients. The partial correlation coefficients of aetiocholanolone with each of the other variables are shown in Table II.

TABLE II.—*Partial Correlation Coefficients of Aetiocholanolone and Variables of Possible Prognostic Significance*

| | | Edinburgh | |
|---|---|---|---|
| | Cardiff (52 patients) | Radical mastectomy (68 patients) | Total group (139 patients) |
| Age | −0·29 | 0·00 | −0·02 |
| Grade | 0·30* | 0·01 | −0·19* |
| Round cell involvement | −0·30* | 0·15 | 0·18* |
| Clinical involvement | 0·11 | 0·02 | 0·01 |
| Histological involvement | 0·12 | 0·02 | — |
| Size | 0·22* | 0·07 | 0·01 |

These coefficients reflect the association between aetiocholanolone levels and each of these variables, after mutual association with menopausal status and with each of the other variables, has been taken into account (Steel and Torrie, 1960). In the Edinburgh series, the full set of partial correlation coefficients could only be obtained for the 68 patients undergoing radical mastectomy, from whom histological examination of nodes was possible.

No consistent correlation was found between aetiocholanolone levels and any of these factors of prognostic significance. Significant but diverging relationships were, however, observed in Cardiff and in the full Edinburgh series with regard to both tumour grade and round cell infiltration. No obvious reasons are apparent for these paradoxical observations.

### DISCUSSION

Using data obtained separately from the 2 centres, it has been possible to define the excretion of aetiocholanolone in control women and patients with benign and malignant breast disease, with respect to their age and menstrual status. By examining the data from patients with early breast cancer, it has also been possible to relate these aetiocholanolone levels to other factors known to be of prognostic significance.

The relationship between urinary aetiocholanolone and age has previously been expressed in the form of a quadratic equation (Cameron *et al.*, 1970), increasing age being associated with decreasing aetiocholanolone excretion. Whilst the present results are in accord with these findings, they also indicate that age *per se* is not associated with lowered aetiocholanolone levels. If the results are re-plotted after dividing the patients into pre- and post-menopausal groups, then age alone fails to influence urinary aetiocholanolone excretion significantly. It seems, therefore, that the reduction in aetiocholanolone observed with increasing age is a result of the increasing proportion of post-menopausal women. This relationship is also observed in control patients and in those with benign breast disease.

The present study, using a large number of patients, confirms the findings of Wade *et al.* (1969) and Cameron *et al.* (1970) that the mean urinary aetiocholanolone levels of patients with early breast cancer are no different from those with either benign disease or in-patient controls. The observation that patients with epitheliosis (believed to be a premalignant condition) have normal aetiocholanolone levels does not support the suggestion that low aetiocholanolone levels may occur in women susceptible to breast cancer.

It has not been possible to demonstrate any consistent relationship between preoperative urinary aetiocholanolone and any single one of a series of clinical and morphological factors of known prognostic significance in women with early breast cancer. These observations, therefore, are at variance with the suggestion that aetiocholanolone is of major prognostic significance in early breast cancer (Hayward and Bulbrook, 1968). In this respect, the time of the urine collection may be of importance. The data in the present study is based upon preoperative specimens whereas those in Bulbrook's study related to urine collected 10 days after mastectomy. It is now known that mastectomy, possibly *via* the stress of surgical procedures, influences both urinary and plasma levels of androgenic steroids (Hayward and Bulbrook, 1968; Wang *et al.*, 1974). Conversely, patients awaiting surgery are likely to suffer pre-operative stress and this may also contribute to differences in pre- and post-operative steroid excretion.

It should be noted, however, that this study shows one group of patients, *i.e.* the post-menopausal women, who are likely to have low urinary aetiocholanolone levels. It is therefore interesting that pre-menopausal women over 35 years with breast cancer have a better prognosis than those women who are post-menopausal (Hamilton *et al.*, 1974). We therefore emphasize the importance of menopausal status in early breast cancer and suggest that it should be taken into account when assessing the possible predictive value of urinary aetiocholanolone.

This work was supported in Cardiff by Tenovus, and in Edinburgh by a grant to Sir John Bruce from the Cancer Research Campaign. The authors wish to thank N. Gleave, H. Stewart, M. M. Roberts and N. Campbell for their help in compiling data for the Cardiff patients. Similar thanks are offered to the surgeons and radiotherapists in Edinburgh who took part in the Edinburgh Breast Trial, and to Mr T. McNair who allowed us to study his patients as a control group. The authors are also grateful to the pathologists in both Cardiff and Edinburgh for their co-operation and generous provision of histological material.

### REFERENCES

ALQUIST, K. A., JACKSON, A. W. & STEWART, J. G. (1968) Urinary Steroid Values as a Guide to Prognosis in Breast Cancer. *Br. med. J.*, i, 217.

ATKINS, H., BULBROOK, R. D., FALCONER, M. A., HAYWARD, J. L., MACLEAN, K. S. & SCHURR, P. H. (1968*a*) Ten Years' Experience of Steroid Assays in the Management of Breast Cancer. *Lancet*, ii, 1255.

ATKINS, H., BULBROOK, R. D., FALCONER, M. A., HAYWARD, J. L., MACLEAN, K. S. & SCHURR, P. H. (1968*b*) Urinary Steroids in the Prediction of Response to Adrenalectomy or Hypophysectomy. *Lancet*, ii, 1261.

BULBROOK, R. D., HAYWARD, J. L. & SPICER, C. C. (1971) Relation between Urinary Androgens and Corticoid Excretion and Subsequent Breast Disease. *Lancet*, ii, 395.

BULBROOK, R. D., HAYWARD, J. L., SPICER, C. C. & THOMAS, B. S. (1962) Abnormal Excretion of Urinary Steroids by Women with Early Breast Cancer. *Lancet*, ii, 1238.

CAMERON, E. H. D., GRIFFITHS, K., GLEAVE, E. N., STEWART, H. J., FORREST, A. P. M. & CAMPBELL, H. (1970) Benign and Malignant Breast Disease in South Wales: A Study of Urinary Steroids. *Br. med. J.*, iv, 768.

CHAMPION, H. R. & WALLACE, I. W. J. (1971) Breast Cancer Grading. *Br. J. Cancer*, **25**, 44.

CHAMPION, H. R., WALLACE, I. W. J. & PRESCOTT, R. J. (1972) Histology in Breast Prognosis. *Br. J. Cancer*, **26**, 129.

HAMILTON, T., LANGLANDS, A. O. & PRESCOTT, R. J. (1974) The Treatment of Operable Cancer of the Breast: A Clinical Trial in the South East Region of Scotland. *Br. J. Surg.*, **61**, 758.

HAYWARD, J. L. & BULBROOK, R. D. (1968) Urinary Steroids and Prognosis in Breast Cancer. In *Prognosis Factors in Breast Cancer*. Ed. A. P. M. Forrest and P. B. Kunkler. Edinburgh: Livingstone. p. 383.

KUMAOKA, S., SAKANCHI, N., ABE, O., KUSAMA, M. & TAKATANI, O. (1968) Urinary 17-Ketosteroid Excretion of Women with Advanced Breast Cancer. *J. clin. Endocr.*, **28**, 667.

MILLER, W. R. & FORREST, A. P. M. (1974) Oestradiol Synthesis by a Human Breast Carcinoma. *Lancet*, ii, 866.

PATEY, D. H. & SCARFF, R. W. (1928) The Position of Histology in the Prognosis of Carcinoma of the Breast. *Lancet*, i, 801.

PATEY, D. H. & SCARFF, R. W. (1929) Further Observations on the Histology of Carcinoma of the Breast. *Lancet*, ii, 492.

SNEDDON, A. (1969) Estimation of Urinary 11-deoxy-17oxosteroids using Isotopically Labelled Internal Standards. *J. Endocr.*, **43**, 487.

STEELE, R. G. D. & TORRIE, J. H. (1960) In *Principles and Procedures of Statistics*. London: McGraw-Hill. p. 277.

THOMAS, B. S. (1965) In *Gas Chromatography of Steroids in Biological Fluids*. Ed. M. B. Lipsett. New York: Plenum Press. p. 1.

THOMAS, B. S. & BULBROOK, R. D. (1964) A Rapid Method for the Estimation of Total 11-deoxy-17-oxosteroids in Urine. *J. Chromatogr.*, **14**, 28.

WADE, A. P., DAVIS, J. C., TWEEDIE, M. C. K., CLARKE, C. A. & HAGGART, B. (1969) The Discriminant Function in Early Carcinoma of the Breast. *Lancet*, i, 853.

WANG, D. Y., BULBROOK, R. D., HERIAN, M. & HAYWARD, J. L. (1974) Studies on the Sulphate Esters of Dehydroepiandrosterone and Androsterone in the Blood of Women with Breast Cancer. *Eur. J. Cancer*, **10**, 477.